# Growth and Financial Outcomes of a Remote Physiologic Monitoring Service for Hypertension in the Primary Care Setting

**DOI:** 10.1007/s11606-026-10384-9

**Published:** 2026-04-17

**Authors:** Riley K. Carroll, Cory P. Coffey, Neeraj H. Tayal, Jodi M. Grandominico, Natalie S. Lee, Jennifer A. Sabatino

**Affiliations:** 1 The Ohio State University College of Pharmacy, Columbus, OH USA; 2 The Ohio State University Division of General Internal Medicine, Columbus, OH USA

**Keywords:** hypertension, primary care, remote physiologic monitoring

## Abstract

**Background:**

Remote physiologic monitoring (RPM) presents a promising solution for hypertension management through efficient blood pressure monitoring outside of the traditional clinic setting utilizing monitors that transmit readings automatically to the healthcare team. Barriers to adoption of RPM include addressing the challenge of managing continuous streams of data, patient capability, and financial sustainability. The impacts of different RPM models on enrollment and financial outcomes are unknown.

**Objective:**

The primary objective of this study was to describe the operationalization and expansion of a pharmacist-led RPM service for hypertension in primary care, highlighting its two phases and describing the financial considerations associated with the program.

**Design:**

Retrospective descriptive study using a data analytics platform, operational logs, and chart review of the electronic health record.

**Participants:**

Patients enrolled in the hypertension RPM service from July 1, 2022, to May 21, 2024.

**Main Measures:**

Number of patients enrolled per month, average duration of enrollment for patients in the program, frequency and categorical distribution of RPM codes billed, average charges submitted per patient enrolled, and average reimbursement per patient enrolled.

**Key Results:**

Phase 1, which took place over 14 months, included 140 patients with average charges submitted of $301.46 per patient and average reimbursement of $96.30. The average enrollment duration was 155 days. Phase 2, which took place over 9 months, included 167 patients, with average charges of $412.93 and average reimbursement of $124.17 per patient. Average enrollment duration was 63 days. The categorical distribution of codes billed differed significantly between the two phases (*p*-value < 0.001).

**Conclusions:**

Using clinic-owned Bluetooth-enabled BP monitors that were reused kept the costs of the program low but may have limited enrollment. Use of vendor-provided cellular-enabled monitors was more costly but may have facilitated additional billing opportunities and program growth. Pharmacists play a central role in delivering RPM services.

## Introduction

Despite the availability of effective medications and evidence-based treatment guidelines, hypertension control remains a persistent challenge, particularly within the primary care setting where hypertension is one of the most common reasons for patient visits^1,2^. Inadequate routine monitoring leads to delays in treatment intensification^3^, particularly for populations with geographic, socioeconomic, or systemic barriers to healthcare^4^. Primary care providers (PCPs) are often managing multiple chronic conditions in time-constrained clinic visits, necessitating prioritization of acute medical issues over chronic disease management^5^. Delays between follow-up visits may hinder timely medication adjustments, a well-documented contributor to therapeutic inertia in hypertension treatment^6^.

Remote physiologic monitoring (RPM) has emerged as a promising solution to address these barriers by allowing continuous monitoring of physiologic parameters outside of traditional in-office visits. Studies have demonstrated that RPM can enhance hypertension management by providing real-time data, enabling timely treatment modifications, and increasing patient engagement^7,8^. Clinical outcomes of RPM interventions for hypertension include significant reductions in systolic and diastolic BP compared to usual care^9^, improved medication adherence and reduced hospital and ED use^10^, and sustained blood pressure (BP) control for up to 42 months post-enrollment^11^. Furthermore, pharmacist-led interventions in medication management and patient education have demonstrated success in improving BP control and adherence^10,12,13^. In response to these data, guidelines and expert statements for hypertension management highlight RPM and a team-based approach to management of the associated data as an important adjunct to usual care.^1,^^14^^,15^

Despite its benefits, widespread adoption of RPM faces several challenges. Management of continuous streams of patient data can lead to provider fatigue if not appropriately integrated into clinical workflows.^16^Additionally, patient technological literacy varies, and ensuring adherence to proper home monitoring practices is crucial for program success^17^. Financial sustainability also poses a concern, as reimbursement models and provider compensation for RPM services continue to evolve^18^.

To address these challenges and improve BP control, a pharmacist-led RPM service for hypertension was developed within a network of primary care clinics affiliated with a large academic medical center in the Midwest^19^. Within this model, embedded clinical pharmacists played a central role in patient enrollment, management of blood pressure data under a collaborative practice agreement (CPA), and facilitation of billing for the service by primary care providers using RPM codes aligning with emerging reimbursement opportunities for digital health interventions^20^. In this report, we outline the development and expansion of this service, detailing patient enrollment trends, billing processes, and reimbursement rates. We share early observations on operationalization and scalability of this primary care pharmacist-led RPM model, contributing to the growing literature on innovative approaches to hypertension management in primary care settings.

## METHODS

### Setting and Participants

This intervention was conducted in Columbus, OH, within a network of seven general internal medicine (GIM) clinics affiliated with a large academic medical center supporting approximately 20,000 adult primary care patients with hypertension, approximately 60% of whom are estimated to have uncontrolled hypertension. During the period of observation, the clinics were staffed by more than 80 attending physicians and 100 medical residents, with clinical pharmacy services provided by nine clinical pharmacists (7.5 full-time equivalents FTEs) and three pharmacy residents. Clinical pharmacists and pharmacy residents operated under CPAs, which authorized them to provide chronic disease management for diverse conditions in collaboration with licensed physicians or advanced practice providers. Under the hypertension CPA, pharmacists were privileged to initiate, discontinue, and adjust antihypertensive therapy, as well as order laboratory monitoring to guide therapeutic decision-making.

### Program Description

Primary care providers identified patients with uncontrolled hypertension and referred them to the embedded primary care pharmacists for medication management and RPM enrollment. At the time of enrollment, the pharmacist consented the patient to RPM and provided them with a blood pressure monitor. In the initial 14-month period from July 2022 to August 2023, which we call Phase 1, the service utilized clinic-owned Bluetooth-enabled blood pressure monitors connected to a smartphone application hosted by the manufacturer. This application transmitted blood pressure data to the patient’s electronic health record (EHR) for provider review following a multistep manual process to establish connection via the patient portal application. Devices were loaned to patients for the duration of their engagement in the program. In Phase 2, starting in September 2023, the program transitioned to a partnership with a third-party vendor that supplied cellular-enabled blood pressure monitors, which transmitted data directly into the EHR, reducing reliance on patient smartphones, expanding eligibility for the program, and enhancing data integration.

Pharmacists enrolled patients either in-office during a scheduled PCP visit or dedicated pharmacist-led visit (Phase 1 and Phase 2) or through pharmacist outreach via telephone (Phase 2 only, as vendor-supported devices could be drop-shipped to patients). During these encounters, the pharmacist consented the patient to the service and provided education on proper blood pressure measurement technique, critical high and low BP values, and appropriate responses to concerning readings. Patients were instructed to monitor their BP daily and were scheduled for a follow-up visit with a pharmacist via telehealth. After documenting the enrollment encounter, pharmacists were responsible for proper reporting of initial patient setup and training, ensuring 16 days of data transmitted from the device before entering CPT code 99453, and routing the encounter to the referring provider for signature. Billing practices were determined in consultation with organization compliance specialists.

Pharmacists engaged with patients regularly through scheduled telehealth encounters and unscheduled outreach in response to alerts to review BP data, assess adherence and tolerability to antihypertensive therapy, and adjust medication regimens as warranted. Pharmacists communicated with PCPs after each patient interaction by routing the encounter within the EHR. While BP data and antihypertensive therapy were primarily managed by pharmacists, collaboration with the PCP occurred when necessary for discussion of complex clinical scenarios or concerns outside of hypertension management. Continuous monitoring and ongoing patient engagement supported timely interventions throughout enrollment, with RPM CPT codes 99454, 99457, and 99458 billed for device supply and remote data transmission, initial treatment management and patient communication, and additional time spent on patient monitoring and engagement per calendar month, respectively. Through a partially automated process, a monthly report of enrolled patients meeting days of data criteria for billing 99454 facilitated appropriate attribution of the code by administrative staff. Pharmacists and PCPs documented time spent engaging in RPM in a specified field within the EHR, which was configured to tabulate the documented time and generate CPT codes 99457 and 99458 as appropriate, helping to ensure consistent and accurate code capture.

**Figure 1:**
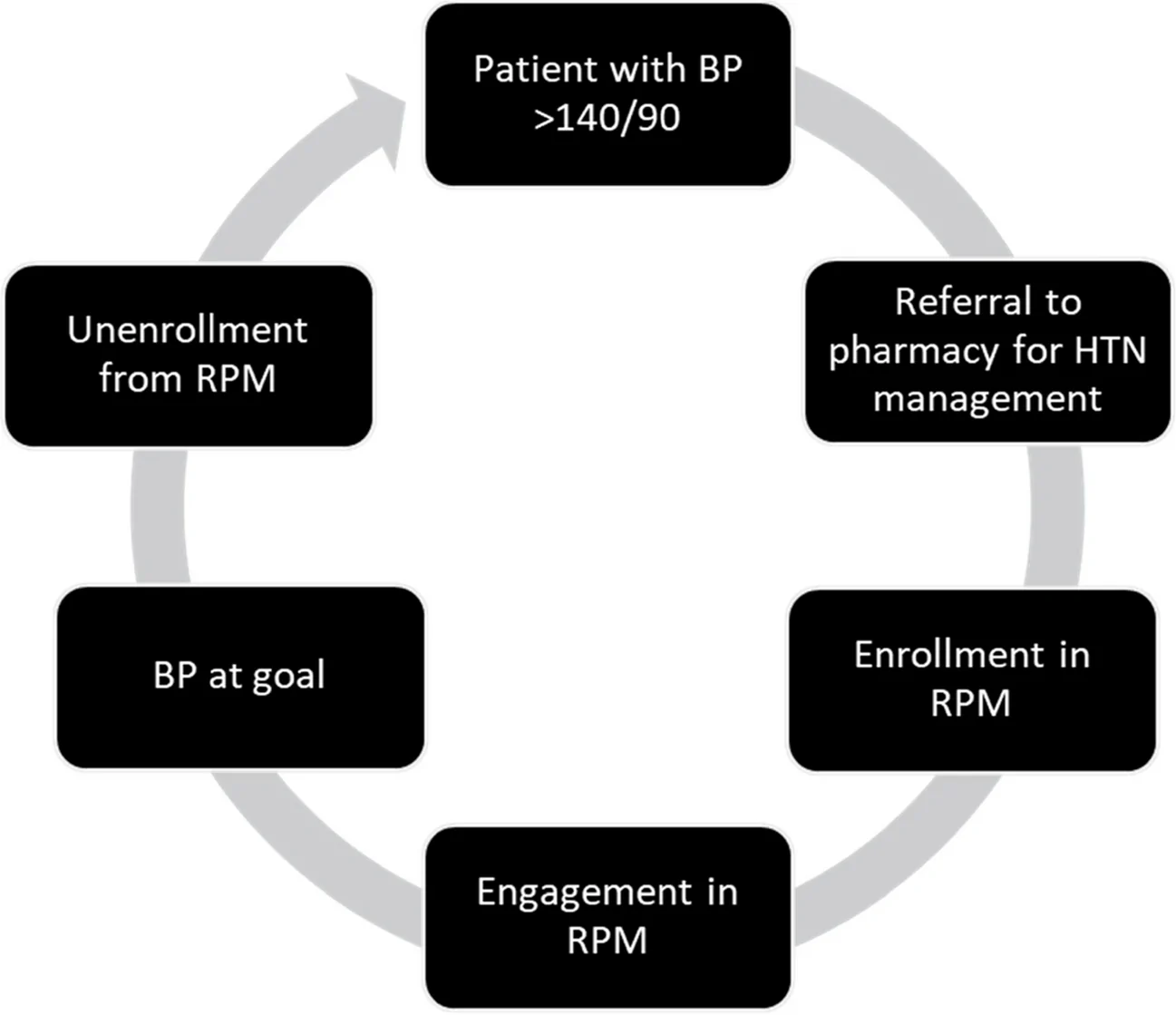
Ideal RPM workflow.

Patients could be unenrolled from the RPM program when their targeted treatment goals were achieved, if they were deemed lost to follow-up, or at any point that the patient or care team decided to end the engagement, at which point long-term hypertension management was transitioned back to the PCP. In Phase 1 of the program, BP devices were returned to the office to be refurbished and re-deployed to a new patient, whereas in Phase 2 patients kept the device and the data transmission from the device was terminated. Patients were able to re-enroll in RPM in the future if BP control deteriorated or additional monitoring was required. The ideal RPM workflow is depicted in Fig. 1.

### Evaluation Methods

Data were collected from a clinical analytics platform integrated with the EHR, operational logs maintained by the pharmacists providing the service for management of the program, and manual chart review. Patients were included if they were enrolled in the hypertension RPM service from July 1, 2022, to May 21, 2024. The following data were collected: patient demographics, consulting prescriber name and practice location, RPM enrollment date, RPM un-enrollment date, charges associated with codes billed, and reimbursement for charges billed. Data were reported using descriptive statistics. Unadjusted differences in the distribution of the codes billed between the two time periods were assessed using a Pearson chi-square test. This study was approved by the Institutional Review Board with a waiver of informed consent and SQUIRE reporting guidelines were used^21^.

## RESULTS

A total of 307 patients participated in the hypertension RPM service within the study period. Characteristics of patients included are shown in Table 1.

**Table 1:** Patient Characteristics

Characteristic	Number of patients (%), *N* = 307
Sex	
Female	162 (52.8)
Race	
White	211 (68.7)
African American/Black	71 (23.1)
Asian	10 (3.3)
Other	15 (4.9)
Coverage type	
Commercial	166 (54.1)
Medicare	105 (34.2)
Medicaid	28 (9.1)
Self-pay	5 (1.6)
Other	3 (1.0)

**Figure 2:**
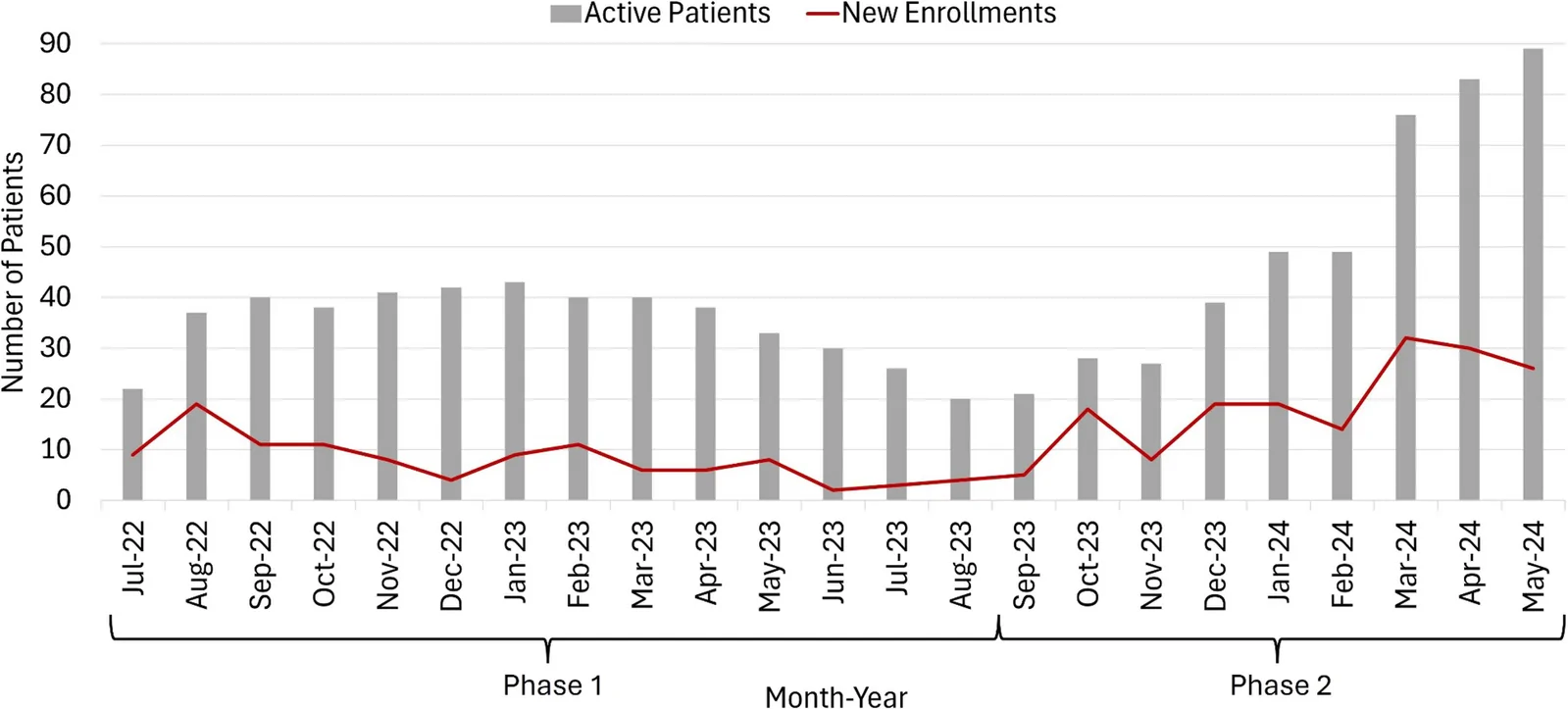
Growth of the hypertension RPM service.

Patient enrollment and active participation in the hypertension RPM program evolved across the two phases (Fig. 2). Early in Phase 1 (July 2022–August 2023), there was a peak in monthly new enrollments which eventually stabilized. The number of active patients during this phase remained relatively stable until decreasing when the transition to Phase 2 began. In Phase 2 (September 2023–May 2024), trends in both new enrollments and active patients increased over time.

Across the two phases, distinct patterns emerged in enrollment and billing activity (Table 2). While the average duration of patient enrollment decreased from Phase 1 to Phase 2 for those enrollments completed within the study period (*N* = 224), a greater total number of patients were enrolled in Phase 2 compared to Phase 1. Financial patterns also evolved over time. Both the average charges submitted and the average reimbursement per enrolled patient increased from Phase 1 to Phase 2.

**Table 2:** Enrollment and Billing in Phase 1 vs Phase 2

	Phase 1	Phase 2
Duration of phase	14 months	9 months
Total number of patients	140	167
Average charges submitted per patient enrolled	$301.46	$412.93
• Total 99453	51	25
• Total 99454	52	155
• Total 99457	108	190
• Total 99458	44	59
Average reimbursement per patient	$96.30	$124.17
Average duration of enrollment	155 days	63 days

**Figure 3:**
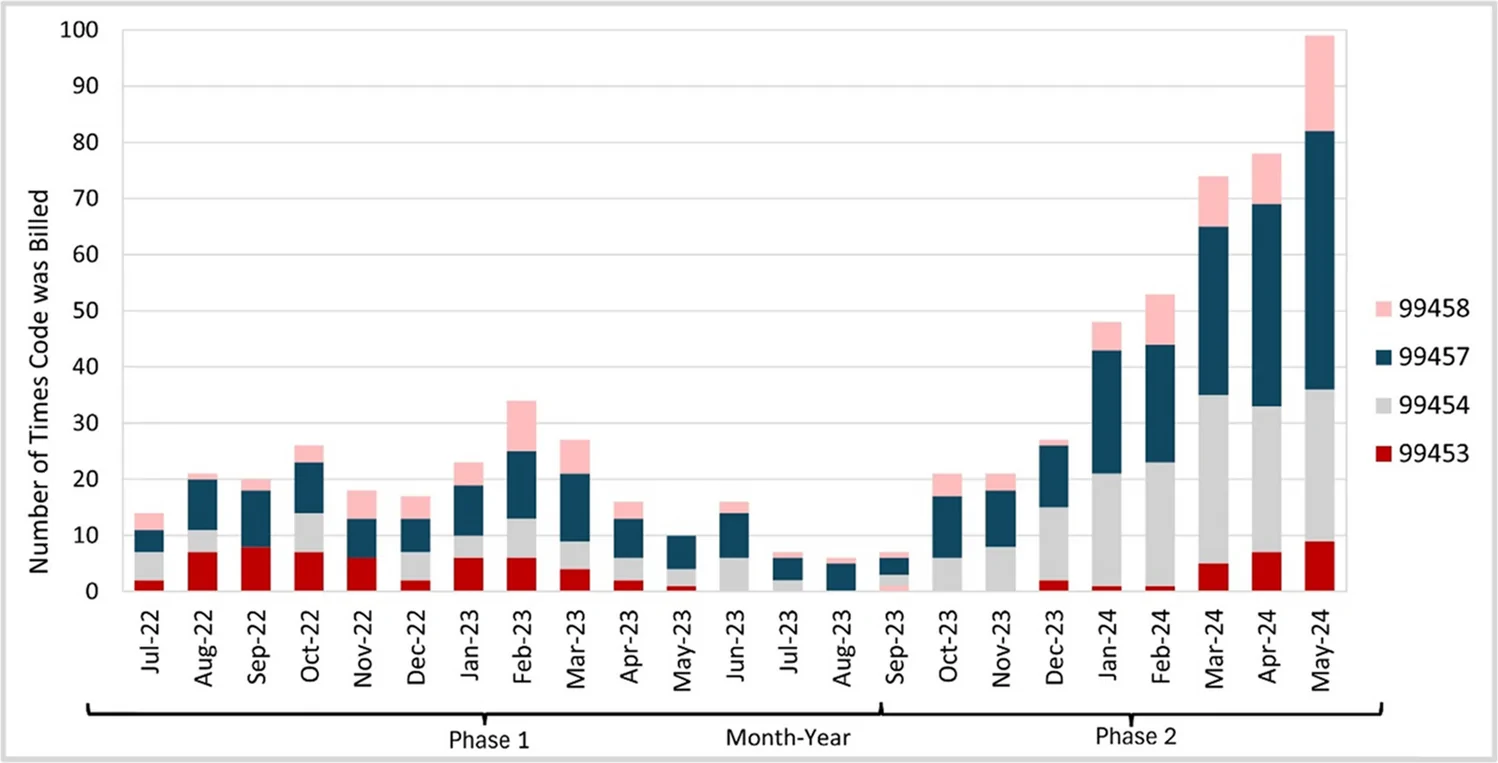
CPT codes billed over time. The categorical distribution of codes differed significantly between the two time periods (*p*-value < 0.001).

Billing activity for RPM services demonstrated an upward trajectory across the two phases (Fig. 3). During Phase 1, billing volumes were relatively modest, with most months having fewer than 30 total code submissions. Changes were also seen in the frequency of CPT codes billed, moving from Phase 1 to Phase 2, with a decrease in 99453 and an increase in 99454, 99457, and 99458 charges submitted. The categorical distribution of codes differed significantly between the two time periods (*p*-value < 0.001), with 99454 (device supply and data transmission) and 99457 (treatment management) emerging as the most common codes billed.

## DISCUSSION

This study describes the operationalization of a pharmacist-led remote physiologic monitoring service for hypertension within a real-world primary care setting. Our results provide key insights for the feasibility and sustainability of pharmacist-led RPM for hypertension in primary care.

Phase 1 relied on clinic-owned Bluetooth-enabled blood pressure monitors, offering several advantages. These monitors were third-party validated for accuracy,^22^ and their refurbishment and reuse kept program costs low. However, significant challenges arose due to their technological requirements. Patients needed a compatible smartphone, had to navigate both the manufacturer’s application and the EHR portal application, and were required to visit the clinic for device deployment. These barriers limited enrollment and frequently led to time-consuming troubleshooting during pharmacist-led visits. Technical issues sometimes resulted in unsuccessful RPM engagement, potentially limiting billing based on existing minimum requirements for days of data transmission and time spent interacting with the patient in management of the data.

The transition to vendor-provided cellular-enabled blood pressure monitors in Phase 2 likely facilitated improvements, as it removed the need for smartphones, broadening patient eligibility and streamlining data transmission. Higher rates of patients were enrolled for shorter durations in Phase 2 compared to Phase 1, and there was a statistically significant difference in the categorical distribution of CPT codes billed. Codes 99454 and 99457 were billed most, demonstrating that patients were successfully transmitting at least 16 days of data in a 30-day period and that the care team was spending at least 20 min of time in RPM treatment management services in a calendar month. The vendor partnership further enhanced efficiency by enabling remote deployment of devices and outsourcing technical support, allowing pharmacists to focus on clinical care rather than troubleshooting technology issues. However, this transition also introduced new challenges, including added program costs such as the increased cost of providing new devices for each patient, occasional patient confusion regarding the vendor’s role, and the need for pharmacists to manually discontinue data transmission through an external platform rather than through the EHR.

Key challenges in RPM implementation include effective management of continuous patient-generated data and costs of RPM services. For RPM to succeed, healthcare providers must incorporate these data streams into their workflows without experiencing burnout or information overload. Leveraging pharmacists and other clinical staff to manage routine monitoring tasks can alleviate provider burden while maintaining high-quality patient care. In the existing reimbursement environment, we found that RPM was a viable avenue for generating revenue through reimbursable services completed by the pharmacists. The utilization of CPT codes 99453, 99454, 99457, and 99458 provided reimbursement for patient enrollment, education, remote monitoring, and telehealth consultations, which contributed to justification of pharmacist time and costs of vendor partnership. Adoption of CPT codes 99445 and 99470 in the Medicare Physician Fee Schedule for 2026 offers lower minimum requirements, expanding reimbursement opportunities.^23^

The scalability of this model further underscores the potential for RPM integration in primary care settings. Despite the program’s expansion, pharmacist FTEs remained unchanged, indicating that RPM can be integrated into existing workflows without significant structural modifications. Moreover, broadening the scope of RPM services to include additional chronic conditions beyond hypertension could further support the financial viability of pharmacist-led care models.

However, sustaining financial success requires adaptability to evolving reimbursement policies. Changes in RPM billing regulations necessitated close collaboration with our compliance department to ensure continued eligibility for reimbursement. At times, billing was temporarily paused during workflow adjustments, leading to potential revenue loss. There was a lower incidence of 99453 billing in Phase 2, which may have been due to the new ability to deploy devices remotely and the organization-specific compliance policy at the time that 99453 could only be billed for face-to-face deployment. Additionally, the need for manual entry of CPT code 99453 after 16 days of data transmission highlighted the inefficiencies of existing billing processes. Fully automating RPM billing within the EHR could optimize revenue capture and ensure that billing opportunities are not overlooked.

This report was not designed to evaluate clinical outcomes or conduct a formal cost-effectiveness analysis. While we reported billing and reimbursement trends, vendor-related expenses were not tracked at the individual patient level and therefore per-patient cost comparisons could not be calculated. Additionally, clinical characteristics of enrolled patients were not included, as the primary aim was to describe operational considerations rather than patient-level outcomes. These factors preclude conclusions about financial sustainability or clinical effectiveness. Future studies should incorporate comprehensive cost data as well as clinical data to more fully assess the impact and scalability of this type of service.

## CONCLUSION

A pharmacist-led hypertension RPM service demonstrated the potential to generate revenue through reimbursable digital health services. The use of clinic-owned Bluetooth-enabled monitors helped control program costs but may have constrained patient enrollment. In contrast, transitioning to vendor-provided cellular-enabled monitors may have facilitated program growth and enhanced billing opportunities. These findings highlight the importance of balancing cost-effectiveness with scalability when designing RPM programs. Pharmacists can play a central role in delivering RPM services, and RPM has the potential to be a financially viable model for pharmacists to optimize chronic disease management while expanding access to care. Further refinement of billing and reimbursement processes is needed, and additional economic modeling will be essential to inform long-term sustainability.

## Data Availability

Data collected for this study are not publicly available due to institutional restrictions. Sharing of data will be considered upon reasonable request and permission from our institution.
